# Developing Clinical Decision (Support) Systems Combining the Scientific and Regulatory Perspective: European Insights on Challenges, Requirements, and Practical Guidance

**DOI:** 10.2196/72809

**Published:** 2026-03-09

**Authors:** Sanne E W Vrijlandt, Sanne W C M Janssen, Annemarie M C van Rossum, Niek B Achten, Rianne Oostenbrink

**Affiliations:** 1 Department of Pediatrics, Division of General Pediatrics, Erasmus MC-Sophia Children's Hospital, University Medical Center, Dr. Molewaterplein 40, Rotterdam, 3015 GD, The Netherlands, 31 (0)10 703 614; 2 Department of Pediatrics, Division of Pediatric Infectious Diseases and Immunology, Erasmus MC-Sophia Children's Hospital, University Medical Center, Rotterdam, The Netherlands; 3 Department of Pediatrics, Erasmus MC-Sophia Children's Hospital, University Medical Center, Rotterdam, The Netherlands

**Keywords:** clinical decision support systems, regulatory requirements, pediatrics, infection diseases, diagnostic reasoning

## Abstract

Clinical decision-making is a critical process where physicians balance risks and benefits. Clinical Decision Support Systems (CDSSs) are increasingly used to help in this process. The regulatory landscape for CDSSs is evolving significantly, with the new European Medical Device Regulation (MDR) now requiring, CE certification for certain CDSSs. This shift poses challenges for health care providers to develop CDSSs in an effective and useful manner while adhering to regulations. This viewpoint comments on diverse challenges and provides solutions to develop a reliable, well integrated and practical tool for clinical use. Using three tools (the Early Onset Sepsis Calculator, Feverkidstool, and Neonatal Procalcitonin Intervention Study algorithm) as examples, we explore the development of CDSSs across four core characteristics: scientific basis, technical aspects, safety, and sustainability. These characteristics recur across the main development processes; scientific development, regulatory assessment, and implementation in routine practice. Successful integration of CDSSs into clinical practice requires a comprehensive understanding of the interconnections between these processes. For example, decisions on algorithm validation and platform selection in the scientific process influence choices for technical safety during the regulatory process. Developers should consider both regulation requirements and clinical needs, to create CDSSs that are not only compliant but also adaptable to the rapidly changing healthcare landscape. We outline a developer’s checklist, for practical guidance, but also appeal for structural support, including national protocols and dedicated hospital roles, to help developers implement CDSSs successfully.

## Juggling Risks and Benefits Using Tools—A Doctor’s Evolving Job

Clinical decision-making involves a delicate balancing act between risks and benefits. It requires the synthesis of a physician’s clinical intuition with objective variables. For instance, deciding whether to start or stop antibiotic treatment in children at risk for severe bacterial infections illustrates this process. Distinguishing between early-stage bacterial illness and viral or physiological causes in neonates and febrile children is particularly challenging, and wrong decisions can have fatal consequences. To aid in making accurate decisions, clinicians may use clinical decision support systems (CDSSs). As these tools become more integral to clinical practice, the regulatory framework governing them is evolving, with significant implications for their development and implementation.

**Figure 1.**
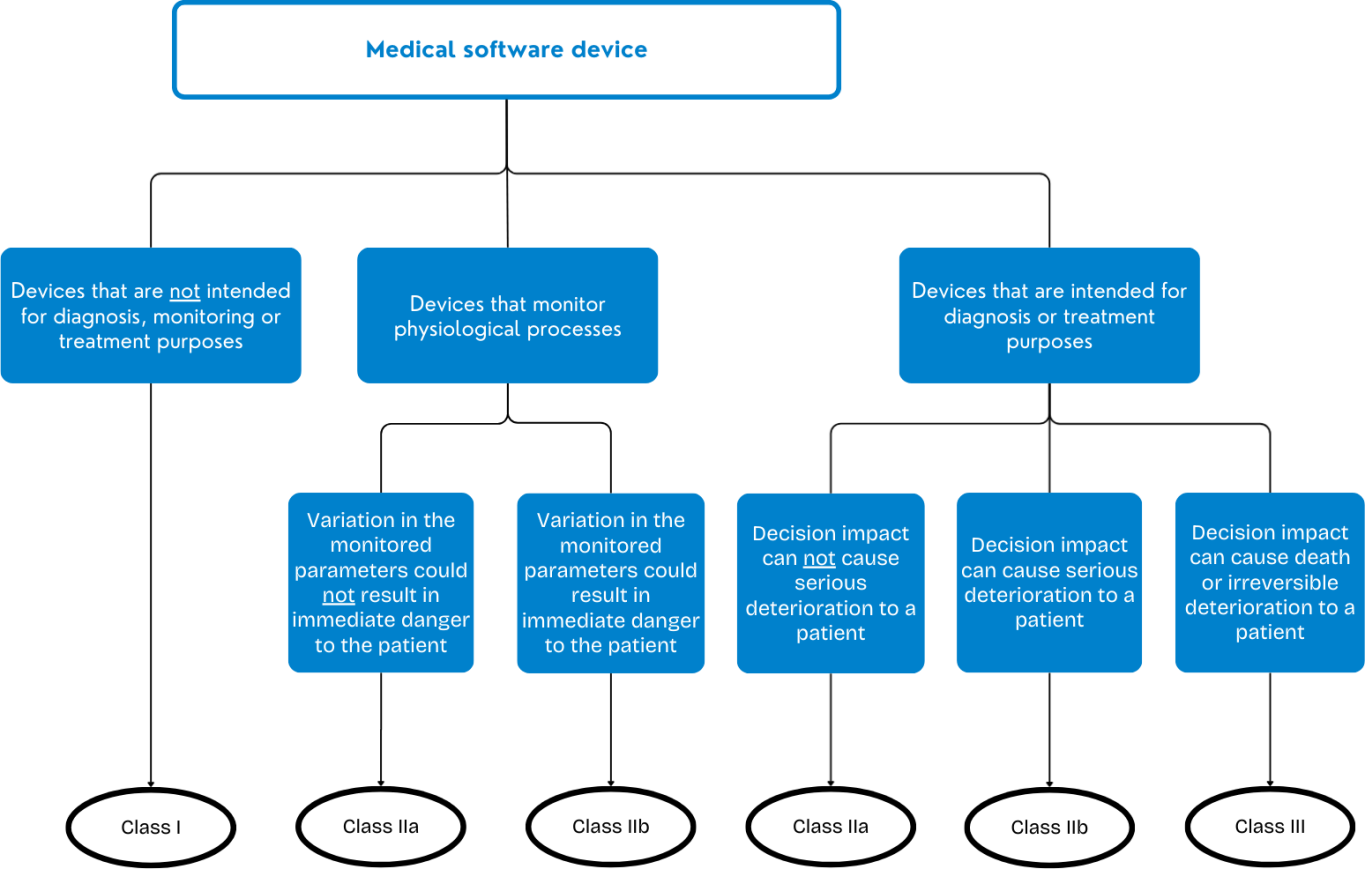
Medical software device classification.

Until 2017, European regulations allowed CDSSs to be used without Conformité Européenne (CE) certification under the Medical Device Directive. However, the introduction of the Medical Device Regulation (MDR) (Textbox 1) expanded the definition of medical devices to include CDSSs [1]. The MDR’s implementation has made CE certification more stringent, linking it directly to a CDSS’s risk classification (Figure 1) [2]. For instance, any CDSS classified from Class II onwards now requires approval from a notified body. Proper classification is the responsibility of the developer. And using only CE-certified CDSSs is the responsibility of the user. Currently, a transition period allows for the legal use of both CE-certified and noncertified CDSSs until 2028. After this period, hospitals and healthcare professionals will bear the responsibility for verifying the certification status of CDSSs used in practice. To aid this verification process, certified devices will be listed in the European Database on Medical Devices (EUDAMED), creating a centralized repository for medical devices used within the European Union.

Textbox 1. **Highlights from the Medical Device Regulation (MDR) regulatory process**

Key changes introduced by the MDR for Clinical Decision Support Systems (CDSSs):

- Risk classification: To ensure safe usage, a 4-scale classification system was added to address appropriate safety measures according to their risk. Additionally, Rule 11 of the MDR regulation states that “software intended tools to provide information which is used to take decisions with diagnosis or therapeutic purposes is classified as class IIa”. As a result, CDSSs are at least classified as class IIa.
- Notified bodies: With more devices falling into higher risk classifications, including CDSSs, more devices need to be revised by notified bodies. Also, stricter requirements account for the approval of the impartial notified bodies.
- Post marketing surveillance: It is mandatory for manufacturers to actively monitor and report on the performance and safety of a device (article 10). This obligation also requires data collection post-implementation ensuring ongoing effectiveness and safety of CDSSs.
- Transparency: The MDR mandates the registration of CDSSs in the European Database on Medical Devices (EUDAMED). This is mandatory to improve insight in which tools are CE-certified by different stakeholders (regulatory authorities, manufacturers, and practitioners).

Successful adoption of CDSSs in routine practice depends on overcoming regulatory and (scientific) developmental hurdles [3]. This viewpoint addresses these obstacles and identifies key improvements needed in the development of CDSSs. While the focus is on the European regulatory environment as regulatory pathways differ across regions, for example, in the United States, where the Food and Drug Administration regulates similar tools under a different framework, some principals may be relevant internationally. Our aim is to support clinical researchers and developers who are at the early stages of CDSS development by providing early insight into the scientific and regulatory considerations that will shape the trajectory of their tool development. This viewpoint synthesizes existing requirements and real-world experiences to clarify key obstacles and areas where alignment is needed. By understanding these challenges early, developers may better anticipate what is required to progress toward successful evaluation and clinical implementation.

## Learning From the Past: Exploring CDSSs With Diverse Characteristics

In this viewpoint we explore three CDSSs designed to assist in the diagnosis and management of pediatric bacterial infections: the Early Onset Sepsis (EOS) Calculator [4], the Feverkidstool (FKT) [5], and the Neonatal Procalcitonin Intervention Study (NeoPInS) algorithm [6]. We described variability in the development and functionality of the CDSSs using previously defined characteristics, including the scientific foundation, the technical design, safety considerations, and sustainability (Table 1) [7].

**Table 1.** Key characteristics of three pediatric clinical decision support systems.

Characteristics of CDSS	Feverkidstool	EOS^a^ calculator	NeoPInS^b^	Regulated by the MDR^c^ [1]
Usage	Helps pediatric ED^d^ physicians assess the risk of serious bacterial infection and bacterial pneumonia in febrile children (>1 mo and <18 y) presenting to the emergency department	Helps doctors and nurses estimate the risk of early-onset neonatal sepsis to guide antibiotic use and clinical management in newborns born at ≥34 weeks	Helps doctors guide the management of suspected early onset neonatal sepsis by supporting decisions on safely discontinuing g antibiotics in neonates born at ≥34 weeks of gestational age	
Scientific basis				
Scientific evidence (highest support)	Stepped-wedge cluster-randomized controlled trial	Meta-analysis and parallel cluster randomized trial	Individually randomized controlled trial	No
Current evidence/validation	(international) external validation	(international) external validation	(international) external validation	No
Reproducibility	In the application a link to the algorithm is provided	In the application a link to the algorithm is provided	No direct link to the algorithm is provided	No
Technical aspect				
CDSS^e^ requirement for algorithm	Tool is required for algorithm	Tool is required for algorithm	A flowchart with reference values is sufficient	No
Platform	Web-based application; accessible through smartphone, tablet, and computer, and integration in the EHR^f^	Webbased application accessible through smart-phone, tablet, and computer, open-source Shiny application, and integration in the EHR	A smart-phone application Print out the flowchart and use it on paper	Yes
Safety				
Accuracy and reliability	Technical assessment	Technical assessment	Superior and noninferior intervention study	Yes
CE-certification	In 2024 under the Medical Device Regulation as a class IIa product	The application in 2021 under the MDD as a class Ia product. The original web-application is not CE-certified^g^ by itself but covered by the EHR certification	In 2021 under the Medical Device Directive as a class Ia product	Yes
CDSS’s final outcome	Risk score with advice	Risk score and prescriptive guidance	Prescriptive guidance	No
Privacy	Data is stored anonymously according to the in ISO13485 and NEN7510-1 mentioned manners	Usage is anonymous, and data is not stored (apart from general storage by using a webpage). If embedded in the EHR, privacy is guaranteed by hospital security systems	Data within the software is stored by the application supplier according to the applicable laws and regulations	Yes
Sustainability				
Funding sources for development and implementation	National funding supported by ZonMW^h^ for development and implementation	Private funding by Kaiser Permanente for development and implementation. EHR implementation is provided by EHR manufacturers.	Private funding by The Thrasher Foundation, The NutsOhra Foundation and the Sophia Foundation for Scientific research for development and implementation	No
Safety monitored during usage	Clinical evaluation report and a post marketing surveillance plan	Clinical evaluation RCT^i^	Clinical evaluation RCT and superior and noninferior intervention study	Yes

a EOS: early onset sepsis.

b NeoPInS: Neonatal Procalcitonin Intervention Study.

c MDR: Medical Device Regulation.

d ED: emergency department.

e CDSS: clinical decision support system.

f EHR: electronic health record.

g CE: Conformité Européenne.

h ZonMW: the Dutch National Health Council.

i RCT: randomized controlled trial.

## Scientific Basis

Across the three CDSSs we examined, the scientific basis emerged as a central yet variably addressed component. The tools differed in the type and strength of evidence underpinning their recommendations. The EOS Calculator mainly draws from meta-analyses of observational studies [8] with a later parallel cluster-randomized trial [9], while NeoPInS and the FKT are based on an individually randomized and stepped-wedge cluster-randomized controlled trials, respectively [6,10].

All three tools included in our analysis underwent international external validation; however, validation strategies can vary considerably, and some CDSSs rely solely on internal validation [3]. Despite the centrality of the scientific foundation, the MDR does not specify requirements for the level of evidence, leaving developers to navigate this crucial aspect independently. Both clinicians and developers should be aware that the MDR procedure does not confirm a tool’s scientific validity.

Transparency also varied across tools. All three publicly display their algorithms in their initial publications [4-6], and both the EOS Calculator and FKT additionally provide algorithm access via their websites, with the EOS Calculator even providing the full algorithm directly on their website. As reproducibility of a CDSS is closely linked to algorithm transparency, this variability may have implications for independent evaluation nevertheless; transparency is not regulated by the MDR.

Finally, the scope plays a significant role in the MDR classification process, as the intended use directly affects the tool’s risk classification (Figure 1). While CDSSs often focus on diagnostic support, as the EOS Calculator, FKT and NeoPINS, their scope can extend beyond diagnosis as they may offer insights into prognosis, treatment, or risk assessment.

Overall, the scientific basis, while not comprehensively covered by MDR regulations, remains critical in CDSS development. Particularly, external validity remains a key consideration, requiring careful attention during development to ensure broad adoption [11]. Moreover, the strength of the scientific foundation and the chosen validation approach influence the market scan and validation processes in the regulatory process.

## Technical Aspects

In our three example tools, we observed substantial variation in how the tools were technically designed and implemented. Platforms ranged from protocol- or paper-based formats to web-based applications, mobile apps, and full integration into electronic health record (EHR) systems. The EOS Calculator and FKT both rely on more complex algorithms, requiring digital platforms, whereas NeoPInS could also be delivered in a protocol or on-paper format. Ultimately, all three tools were developed into applications to support broader clinical use. These differences illustrate how algorithm complexity and intended use shape the technical form a CDSS can realistically take.

Protocol or on-paper tools, such as NeoPInS can be embedded within clinical protocols, offering continuous access at low cost. However, they face challenges in scalability and ease of use, as manual calculations are often required. The responsibility for updates and modifications lies with individual hospitals or local departments. Importantly, on-paper tools are not directly subject to the MDR certification requirements as their use does not necessitate an additional CE-mark.

Web-based applications, such as the EOS Calculator and FKT benefit from global accessibility and ease of integration in EHRs with hyperlinks. However, reliance on web-based platforms introduces vulnerabilities like downtime and security concerns. The MDR places the certification responsibility on developers, necessitating a representative for CDSSs used within the EU but developed elsewhere [1]. The complexity increases when web-based tools developed outside the EU, such as the EOS Calculator web-based version, are accessed from inside the EU.

Mobile apps like the NeoPInS (deprecated) and EOS Calculator app (currently for research purpose in the Netherlands), offer tailored user experience on devices like smartphones or tablets. Although apps provide tailored interactions, they are costly to develop and maintain, requiring agreements on intellectual property and updates.

EHR system integration, as seen with the EOS Calculator and FKT, offers user-friendliness by aligning with existing healthcare infrastructure. While cost-effective, integration across diverse EHR systems can be complex due to the lack of a universal standard. Maintenance typically falls under the purview of EHR providers, but algorithm updates may require prior agreements as they are not automatically managed by the provider.

Taken together, our comparison shows that technical design decisions, shaped by algorithm complexity, workflow integration needs, and resource constraints, directly influence reproducibility, long-term sustainability, and regulatory obligations. Because these factors determine whether a CDSS can be reliably adopted, maintained, and scaled, they must be considered early in the development process rather than treated as secondary concerns.

## Safety

Our comparison shows that safety considerations are shaped not only by the underlying data flows but also by how directive a CDSS‘s recommendation is. Although both the FKT and EOS Calculator generate risk scores, the EOS Calculator offers more prescriptive guidance compared to the FKT. The NeoPInS, without a risk score, also provides prescriptive recommendations. These functional differences influence potential clinical risk. However, although the MDR considers intended scope for classification, it does not account for recommendation type.

Compliance with privacy regulations is mandatory, as CDSSs often handle sensitive patient data. Adherence to the MDR ensures comprehensive coverage of data privacy requirements. Privacy responsibilities also differ by platform: for the EOS Calculator and FKT embedded within an EHR, hospital security systems guarantee privacy compliance, whereas for the web applications of the three tools, the developer ensures it.

Beyond privacy, safety also depends on accuracy and reliability; this involves the technical correctness of a tool’s output and its scientific validity. A reliable CDSS consistently produces accurate results, as demonstrated by the validation in our examples. Risk assessment and mitigation strategies address potential issues such as misdiagnosis or cybersecurity threats. Developers can implement security measures like dual authentication to safeguard CDSSs against cyber threats. However, user account requirements and time-consuming login processes, as seen with the NeoPInS and FKT applications, may hinder adoption and have even led to discontinuation of the NeoPInS app.

Overall, safety is paramount for CDSSs, ensuring that these tools benefit patients without causing harm. Safety is not a single requirement but an interplay of privacy protection, accuracy, reliability, and risk mitigation, all of which require continuous monitoring throughout a CDSS’s lifecycle.

## Sustainability

The sustainability of a CDSS relies on its integration into daily practice, maintenance costs, and available funding. Funding sources vary, ranging from private investors to governmental organizations and research institutions. The funding party’s priorities can influence platform choice, impacting long-term sustainability. In CDSS development, project-based funding is common, posing challenges for continued financial support once projects conclude.

Integration into the EHR, as seen for both the FKT and EOS calculator, can enhance sustainability by facilitating routine use and reducing the need for parallel systems. At the same time, embedding a CDSS within an EHR may introduce challenges related to ownership and governance, as responsibility for the tool, its maintenance, and updates can become less transparent once it is no longer managed as a standalone application. These issues are particularly relevant in the context of MDR requirements, for example post-market surveillance, which mandates ongoing monitoring and data collection after implementation. For EHR-integrated CDSSs, it may be unclear whether such responsibilities lie with the original developer, the EHR vendor, or the health care organization, potentially complicating compliance and long-term accountability.

## Leveraging the Process of Developing Scientifically Valid, Regulatory Proven, and Sustainable CDSSs for Clinical Practice

**Figure 2.**
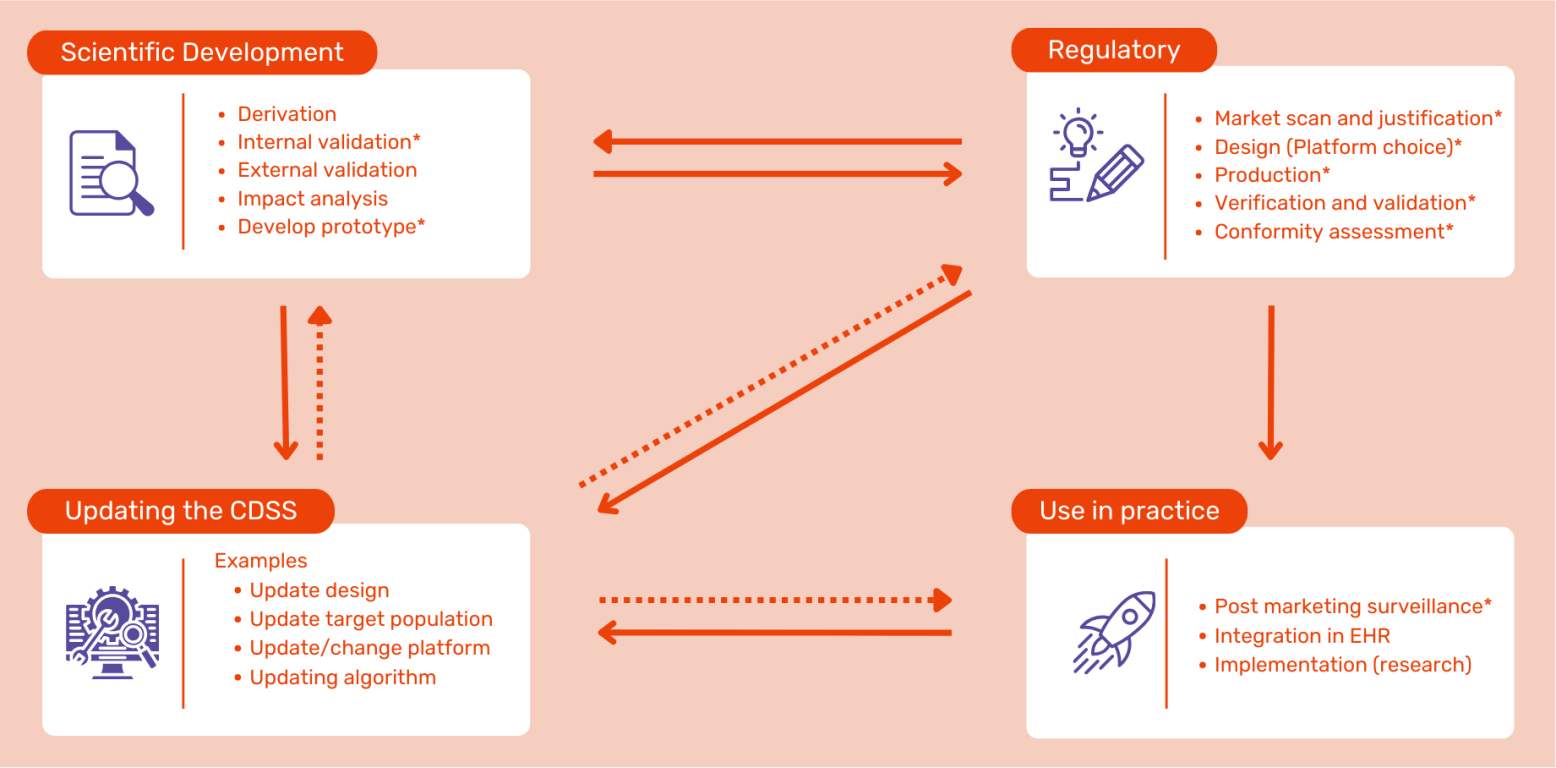
Processes in the clinical decision support system development. CDSS: clinical decision support system; EHR: electronic health record. *Aspects part of the Medical Device Regulation are marked with an asterisk.

From the above, we argue that the developmental processes involved integrating CDSSs into clinical practice should not be portrayed as three consecutive phases but are dynamic and interconnected in practice (Figure 2). Also, these processes are crucial in determining how effectively a CDSS can be integrated into clinical practice, ensuring that it is both reliable and useful for healthcare professionals. This requires developers to navigate them with a strategic approach. Understanding the interplay between these processes is crucial for creating CDSSs that are scientifically sound, regulatory-compliant, practically effective, and ultimately sustainable. While the MDR addresses certain aspects of these processes (eg, internal validity, market scan and justification and post marketing surveillance) it does not comprehensively regulate every characteristic (Table 1), leaving gaps that require careful consideration by developers and clinicians. To translate the lessons learned from the three CDSSs into actionable guidance, we therefore synthesized our observations into a developer’s checklist (Textbox 2), which highlights key considerations across scientific development, technical design, safety, and sustainability.

Textbox 2. **Developer’s checklist for a Clinical Decision Support System (CDSS).**

1. Select the technical platform early and ensure it aligns with all integration and implementation requirements.
2. Engage and communicate with end-users early and throughout the development and design of the CDSS to ensure clinical workflow fits.
3. Ensure the tool’s guidance matches user expectations (e.g. if it provides advice rather than full decision-making, clarify this to avoid overreliance).
4. Plan for both internal validation and external real-world testing.
5. Integrate privacy and security requirements from the earliest stage to avoid delays later.
6. Realistically estimate Conformité Européenne certification workload, expertise, and timelines.
7. Define clear ownership and a sustainable long-term maintenance plan.
8. Prepare fallback strategies to manage tool downtime safely.
9. Keep access simple (e.g. avoid unnecessary login steps).
10. Ensure full integration with existing hospital and electronic health record systems.

A critical, yet often overlooked, aspect of CDSS development is the early involvement of end-users in the scientific development process, as this can improve both tool design, effectiveness, and facilitate smoother transitions to later stages. Early strategic choices, such as selecting an appropriate technical platform, also have important downstream consequences for validity assessment and future usability. For example, although the EOS Calculator was initially developed as a web-based tool to maximize accessibility, this choice later complicated retrospective validation due to the need for manual data handling.

Validation should therefore be planned as a comprehensive, iterative process, combining internal testing with external, real-world evaluation, while accounting for the workload, expertise, and timelines required for CE certification. Beyond validation, long-term success depends on clear ownership, sustainable maintenance strategies, and predefined fallback procedures to ensure patient safety during tool downtime. Practical design considerations, such as minimizing burdensome login procedures and enabling seamless integration with existing hospital and EHR systems, further support adoption and routine use. Addressing these elements across scientific development, regulatory assessment, and clinical implementation processes enhances not only the reliability of a CDSS but also its lasting impact in clinical practice. Although all three CDSSs underwent these three key processes, their approaches differed (Table 1). As illustrated by the publication dates of manuscripts on development, the processes were timed sequentially from which important lessons were learned regarding the need for earlier and more continuous alignment.

A limitation of this viewpoint is that it focuses primarily on European MDR regulations nevertheless, similar challenges have been observed in other regions of the world. Also, currently novel AI techniques, performing the functions of CDSSs, such as dynamic risk prediction and providing targeted clinical advice, are often not yet recognized as CDSSs by clinicians. As a result, these tools are used without formal consideration of their regulatory status, posing challenges to the current MDR regulation.

This viewpoint highlights that the complexity of CDSS development currently places a disproportionate burden on individual developers. While foresight and adaptability remain essential, developers cannot be expected to navigate scientific validation, regulatory compliance, and practical implementation alone. To support clinical scientists, our developer’s checklist (Textbox 2) provides concrete guidance for successful CDSS development. Beyond these practical recommendations, structural changes are needed at the system level, for example, the creation of national protocols, standardized regulatory guidance, and dedicated support structures within hospitals. The MDR provides an important safety framework, but realizing the full potential of CDSSs requires both practical guidance for developers and systemic changes to ensure that these tools can be safely, efficiently, and effectively implemented to support clinicians and improve patient outcomes.

## Abbreviations

CDSSs: clinical decision support systems
CE: Conformité Européenne
EHR: electronic health records
EOS: Early Onset Sepsis
EUDAMED: European Database on Medical Devices
FKT: Feverkidstool
JMIR: Journal of Medical Internet Research
MDR: Medical Device Regulation
NeoPInS: Neonatal Procalcitonin Intervention Study
